# Subpopulations of fibroblasts derived from human iPS cells

**DOI:** 10.1038/s42003-024-06419-8

**Published:** 2024-06-18

**Authors:** Takashi Kobayashi, Akihiro Yamashita, Noriyuki Tsumaki, Hideto Watanabe

**Affiliations:** 1 Institute for Molecular Science of Medicine, Aichi Medical University, Aichi, Japan; 2 Department of Tissue Biochemistry, Graduate School of Medicine and Frontier Biosciences, Osaka University, Osaka, Japan

**Keywords:** Cell biology, Inflammation

## Abstract

Organ fibrosis causes collagen fiber overgrowth and impairs organ function. Cardiac fibrosis after myocardial infarction impairs cardiac function significantly, pulmonary fibrosis reduces gas exchange efficiency, and liver fibrosis disturbs the natural function of the liver. Its development is associated with the differentiation of fibroblasts into myofibroblasts and increased collagen synthesis. Fibrosis has organ specificity, defined by the heterogeneity of fibroblasts. Although this heterogeneity is established during embryonic development, it has not been defined yet. Fibroblastic differentiation of induced pluripotent stem cells (iPSCs) recapitulates the process by which fibroblasts acquire diversity. Here, we differentiated iPSCs into cardiac, hepatic, and dermal fibroblasts and analyzed their properties using single-cell RNA sequencing. We observed characteristic subpopulations with different ratios in each organ-type fibroblast group, which contained both resting and distinct ACTA2^+^ myofibroblasts. These findings provide crucial information on the ontogeny-based heterogeneity of fibroblasts, leading to the development of therapeutic strategies to control fibrosis.

## Abstract (web summary)

Transcriptome analysis of iPSC-derived fibroblasts reveals crucial information on the ontogeny-based heterogeneity of fibroblasts, potentially leading to the development of therapeutic strategies to control fibrosis.

## Introduction

Fibroblasts are mesenchymal cells that form the extracellular matrix (ECM), which supports an organ-specific shape and provides the environment for parenchymal cells^1,2^. The ECM consists of collagen fibers, elastic fibers, and other fibrous components, as well as proteoglycans and glycosaminoglycans that fill the space among the fibers; it provides nutrients, stores cytokines and growth factors, forms their concentration gradients, and regulates cell behavior.

Fibroblasts can be defined as resting fibroblasts, activated/proliferating fibroblasts, and myofibroblasts, depending on conditions. Under physiological conditions, a few quiescent resting-type fibroblasts are present in abundant stroma. Once activated under the inflammation, the resting fibroblasts proliferate and differentiate into active contractile cells, termed myofibroblasts. Transforming growth factor-β (TGFβ) drives the differentiation of fibroblasts toward myofibroblasts and facilitates their biosynthesis of collagens and proteoglycans^3,4^. Myofibroblasts further differentiate into “mature” myofibroblasts, which synthesize collagens more actively. Then, collagen fibers are correctly aligned as myofibroblasts decrease. An imbalance between tissue degradation and repair causes increased volumes of the ECM, leading to fibrosis^5^. Therefore, fibroblasts are directly involved in these pathogenesis and disease progressions.

Fibroblasts are the key players in the development of organ fibrosis, the uncontrolled growth of collagen fibers, leading to organ dysfunction. Cardiac fibrosis after myocardial infarction impairs cardiac function significantly, pulmonary fibrosis reduces gas exchange efficiency, and liver fibrosis disturbs the natural function of the liver. Although the initial stages of fibrosis likely share the same process as tissue repair, persistent activation of fibroblasts by repeated or prolonged stimulation leads to fibrosis.

Fibrosis has organ specificity, defined by the heterogeneity of fibroblasts. For example, systemic sclerosis gradually develops in the skin, esophagus, lungs, and heart but seldom in the liver^6^. Keloid is a continuously growing local fibrotic mass, and such a mass is not observed in other organs^7^. This heterogeneity is known to be established during the embryonic period^8–10^.

Fibroblasts have various origins at different developmental stages^2^. Some fibroblasts are transformed via the epithelial-mesenchymal transition (EMT), in which epithelial cells lose their unique molecular markers, such as E-cadherin or zona occludens-1, and express fibroblast marker proteins such as fibroblast-specific proteins^11^. Other fibroblasts are of hematopoietic stem cell origin. The multipotent progenitor cells can differentiate into various vascular and mesodermal cells, such as pericytes, adventitial cells associated with the vasculature, and mesenchymal stem cells of the bone marrow. They can contribute to the adult interstitial fibroblast population^2,12,13^. The heterogeneity of fibroblast origin, evidenced by the diversity of transcriptome, signal transduction pathways, expression of the ECM molecules, cytokines, and growth factors, determines the stromal property of individual organs and characterizes diseases accompanied by stromal reactions^14–16^. Although fibroblast heterogeneity is believed to underlie disease characteristics, their ontogeny-based processes of heterogeneity formation remain unknown.

Currently, techniques for differentiating induced pluripotent stem cells (iPSCs) into various parenchymal cells, including cardiomyocytes, neurons, and hepatocytes, have been established, and protocols for differentiation into accompanying fibroblasts have been reported. Here, we prepared iPSC-derived fibroblasts in differentiation lineages of the heart, skin, and liver and analyzed their expression profiles using single-cell RNA-sequencing (scRNA-seq). Our results indicate characteristic subpopulations of fibroblasts of each organ type, although they share common features. We classified these subpopulations into active ACTA2^+^ and resting ACTA2^−^-type fibroblasts and identified TBX20 and TRPS1 as common factors in the ACTA2^+^ subpopulations. Our results provide information on organ-specific fibroblast subpopulations and a basis for specific fibrotic conditions in different organs.

## Results

### Differentiation of hiPSCs to fibroblasts

**Fig. 1:**
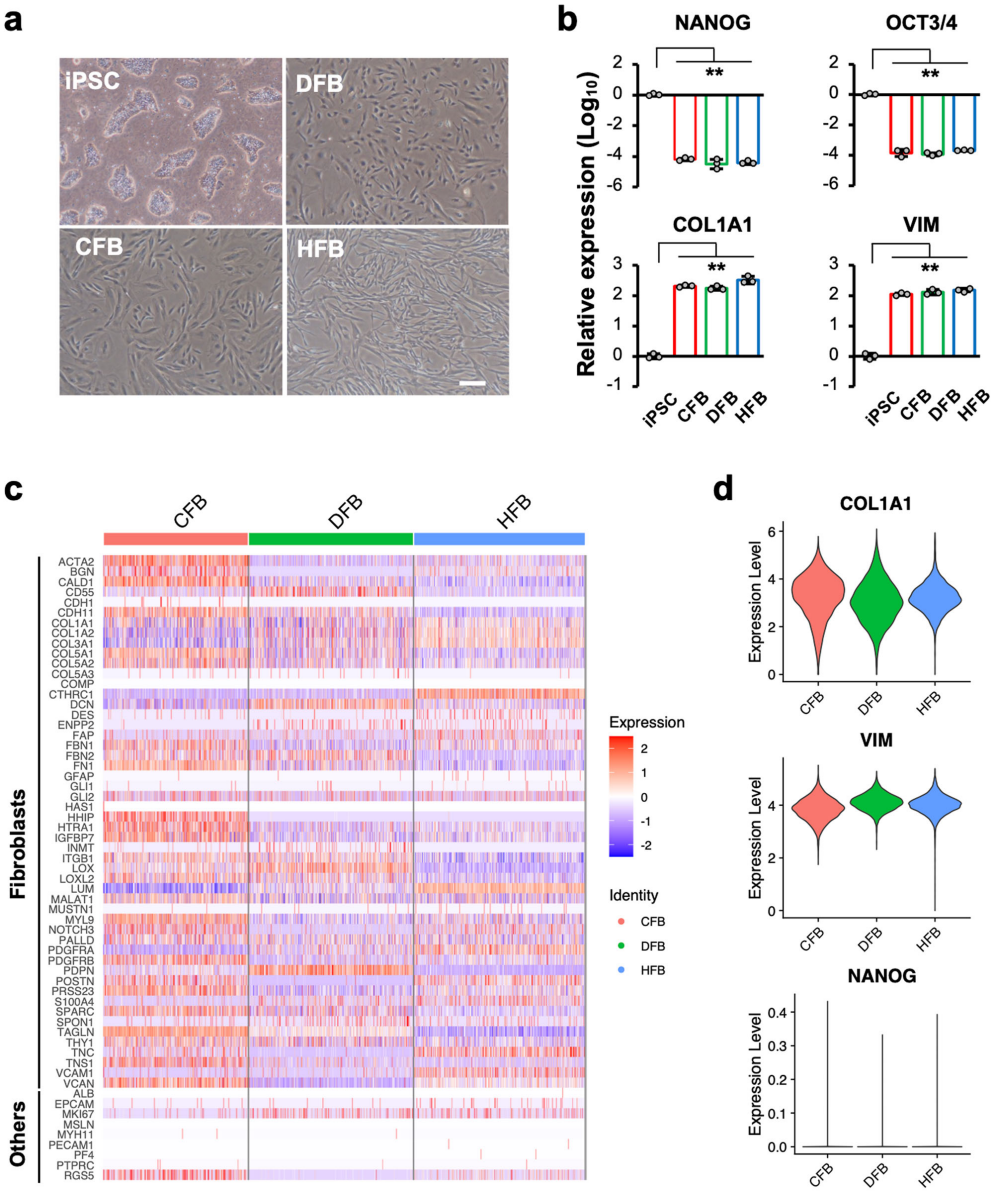
Differentiation of fibroblasts from human iPSC. **a** Phase-contrast views of iPSC and differentiated fibroblasts. DFB dermal fibroblasts, CFB cardiac fibroblasts, HFB hepatic fibroblasts. Scale bar indicates 200 µm. **b** Expression levels of stem markers, *NANOG* and *OCT3/4*, and fibroblast markers, *COL1A1* and *VIM*, in iPSC and differentiated fibroblasts. Data represent the mean ± SD of three independent experiments. One-way ANOVA followed by Dunnett’s multiple comparison test was used to compare the differentiated group with iPSC. **p < 0.01. **c** Heatmap analysis of integrated scRNA-seq data from differentiated fibroblasts. Selected fibroblasts and other lineage marker genes are shown. **d** Violin plots indicate the expression levels of *COLA1A*, *VIM*, and *NANOG* in differentiated fibroblasts.

Normal human iPSCs were differentiated into fibroblast-like skin-(DFB), heart-(CFB), and liver-type (HFB) cells as models for organ-specific fibroblasts (Supplementary Fig. 1). The differentiated cells exhibited spindle-shaped, fibroblast-like morphology (Fig. 1a). Quantitative RT-PCR (qRT-PCR) analysis showed significantly decreased expression of stem cell marker genes, such as *NANOG* and *OCT3/4*, and increased expression of fibroblast marker genes, such as vimentin *(VIM*) and *COL1A1* in differentiated cells compared with undifferentiated iPSCs (Fig. 1b). To investigate the heterogeneity of fibroblasts, we performed transcriptome analysis using these cells as samples for each organ-specific fibroblast model.

### Overview of transcriptome analysis

Single-cell RNA-seq (scRNA-seq) data were obtained from three fibroblast models and then integrated using Seurat, resulting in a total of 15,804 cells (DFB, 5515 cells; CFB, 4490 cells; HFB, 5799 cells). First, we compared the expression of fibroblast marker genes among the three fibroblast models and found that most marker genes, such as *COL1A1* and *PDGFRA*, were expressed in all types (Fig. 1c, d, and Supplementary Fig. 2). In contrast, the expression of other cell marker genes, such as *ALB* (hepatocytes), *GLI1* (neurons), *MYH11* (smooth muscle cells), *MSLN* (enterocytes), *PECAM1* (endothelial cells), *PF4* (megakaryocytes), and *PTPRC* (immune cells), was substantially low. The expression of *RGS5* (pericytes/smooth muscle cells) was observed at a high level in CFB, and *EPICAM* (epithelial cells) was partially observed at low levels in the three fibroblast models. The expression patterns of fibroblast marker genes differed slightly among the models (Fig. 1c and Supplementary Fig. 2). The expression levels of *BGN*, *POSTN*, and *TNC* were markedly lower in DFB (Fig. 1c and Supplementary Fig. 2). *ACTA2*, a myofibroblast marker, was expressed in all models; however, its expression was higher in CFB (Supplementary Fig. 2). The gene expression levels were also confirmed by qRT-PCR (Supplementary Fig. 2b). These differential gene expression patterns demonstrate that the three fibroblast models had different characteristics.

To ensure data reliability, we compared scRNA-seq data from these iPSC-derived fibroblasts with data from three primary human fibroblasts in public databases, PCS-201-012 (adult skin fibroblasts, 6775 cells, GSM5104820)^17^, BJ (neonatal skin fibroblasts, 6024 cells, GSM6894025)^18^, and CCD-18Co (neonatal colon fibroblasts, 2343 cells, GSM6894025)^19^, as well as a dataset from fifteen human organs including heart, skin, and liver (total 84,363 cells, GSE159929)^20^ (Supplementary Fig. 3). Cluster analysis and identification of cell types by marker gene expression patterns revealed that the iPSC-derived fibroblasts were different from organ fibroblasts and other cell types, such as endothelial cells and smooth muscle cells (Supplementary Fig. 3a and Supplementary Data 1)^20^. The iPSC-derived fibroblasts and three primary fibroblasts had very similar cluster compositions, consisting mainly of FB-a (cluster 0), FB-c (cluster 9), and a mitotic cell population (cluster 7) (Supplementary Fig. 3b and Supplementary Data 2). Organ fibroblasts, on the other hand, were dominated by FB-b (cluster 1). However, FB-a and FB-c were detected as major populations in blood, lymph nodes, marrow, small intestine, and spleen, whereas minor populations in the bladder, common bile duct, esophagus, heart, muscle, rectum, skin, stomach and trachea. Taken together, the overall gene expression levels of cultured fibroblasts may be different from in vivo organ fibroblasts.

### Identification of subpopulations

**Fig. 2:**
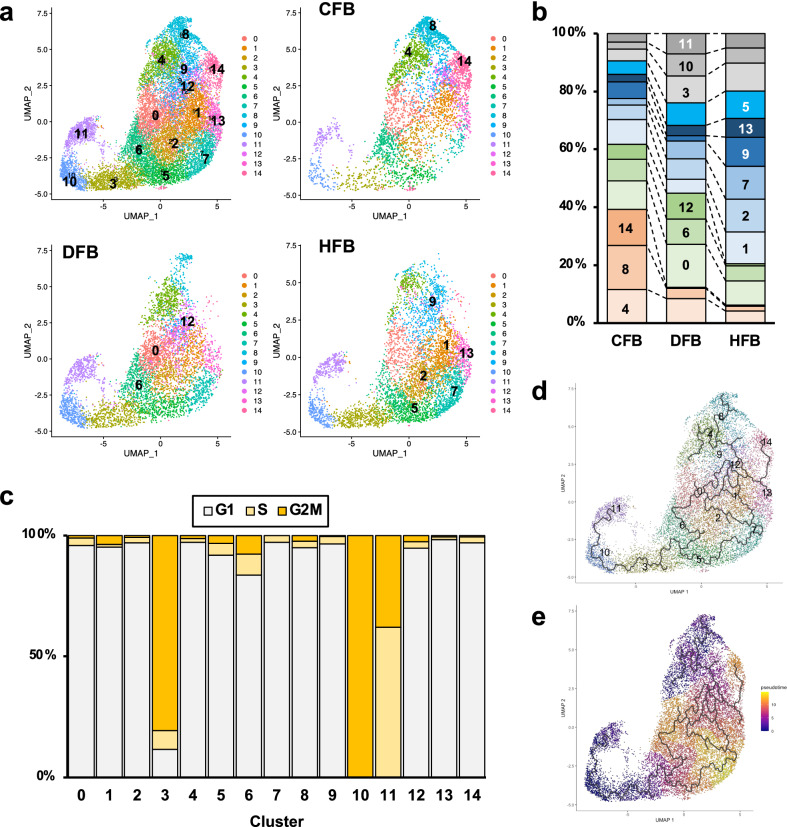
Analysis of scRNA-seq data of the differentiated fibroblasts. **a** The Uniform minifold approximation and projection (UMAP) plot analysis of absolute data and separate data of scRNA-seq. **b** Bar graph showing the proportion of clusters in each sample. The Numbers inside the bars indicate the cluster numbers. **c** Bar graph showing the percentage of cells in each cell cycle phase. **d** Trajectory analysis using the Monocle 3. **e** Pseudotime analysis using the Monocle 3.

To investigate the heterogeneity of fibroblasts, we further analyzed scRNA-seq data from the iPSC-derived fibroblasts. Clustering of the integrated scRNA-seq data to identify fibroblast subpopulations resulted in 15 clusters (Fig. 2a). The proportion of each cluster differed among types (Fig. 2b and Supplementary Table 1). When we examined the cluster characteristics of each cell type, CFB had a high percentage of Clusters 4, 8, and 14, with 39.3% of CFB. In Cluster 14, DFB and HFB comprised less than 0.5% of the cells in each cluster, and CFB comprised almost all the cells in Cluster 14. HFB had a high percentage of Clusters 1, 2, and 7, accounting for 33.6% of HFB. Clusters 9 and 13 were relatively high in HFB (these five clusters together accounted for 50.1% of the total). DFB had a high percentage of Clusters 0, 3, 6, and 12, but since Cluster 3 was assumed to be proliferating cells, as described below, the characteristic clusters of DFB were Clusters 0, 6, and 12, which accounted for 32.5% of DFB. These results suggested that fibroblasts differentiated from iPSCs using different methods have their own major subpopulations. Except for Cluster 14, all clusters consisted of three types, although their proportions differed among the clusters.

**Fig. 3:**
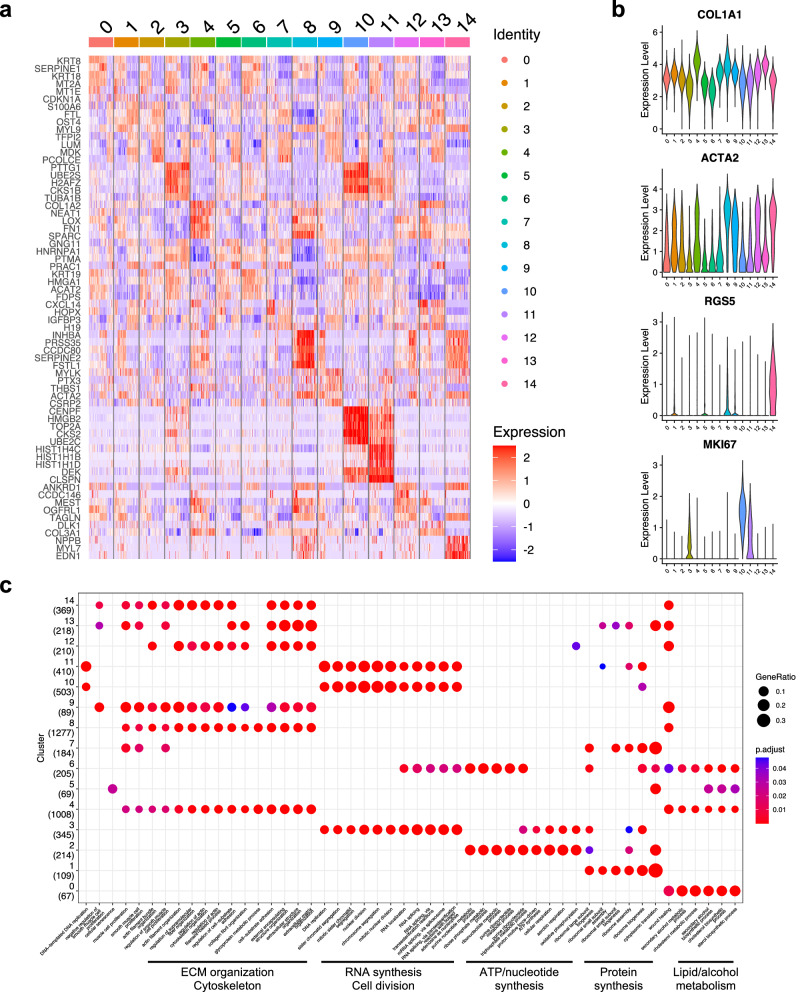
Features within each fibroblast subpopulations. **a** Heatmap showing the top five differentially expressed genes (DEGs) within each cluster. The complete data are presented in Supplementary Data 3. **b** Violin plots indicate the expression levels of *COLA1A*, *ACTA2*, *RGS5*, and *MKI67* in the clusters. **c** Gene ontology enrichment analysis of biological processes in each cluster. The complete data are presented in Supplementary Data 4.

The expression patterns of the characteristic genes in each cluster are shown in a heatmap (Fig. 3a, Table 1, and Supplementary Data 3). Fibroblast marker genes such as *COL1A1* were expressed in all clusters (Fig. 3b and Supplementary Fig. 4), confirming that all clusters were fibroblast populations. Regarding the other cell markers, the expression of *RGS5* was higher in Cluster 14 than in the other clusters, whereas the expression of *MKI67* was higher in Clusters 3, 10, and 11, indicating that these were proliferating cell populations (Fig. 3b and Supplementary Fig. 4). Fibroblast marker gene expression was relatively higher in Clusters 4 and 8. The expression patterns of several marker genes tended to be slightly different in each cluster, suggesting that they were subpopulations. For instance, *LUM* expression was higher in Clusters 2, 7, and 13 (Supplementary Fig. 4). The expression patterns of collagen genes were similar among the clusters, and the overall expression levels were high in Clusters 4, 8, and 13 (Supplementary Fig. 5). The expression levels of some collagen genes were slightly different among the clusters. For instance, *COL14A1* was high in Clusters 7 and 13, and *COL8A1* and *COL9A3* were high in Clusters 8 and 14 (Supplementary Fig. 5).

**Table 1:** List of top ten differentially expressed genes (DEGs)

Cluster(s)	Top 10 DEGs (ave_log2FC)									
0	KRT8	SERPINE1	KRT18	MT2A	MT1E	KRT19	PHLDA2	EZR	PCP4	TGM2
1	CDKN1A	S100A6	FTL	OST4	MYL9	BAMBI	SELENOM	SERF2	SNHG29	CTHRC1
2	S100A6	TFPI2	LUM	MDK	PCOLCE	FTL	BAMBI	GSTP1	CEBPD	GAPDH
3	PTTG1	UBE2S	H2AFZ	CKS1B	TUBA1B	HMGB1	TUBB4B	CDKN3	STMN1	HMGN2
4	COL1A2	NEAT1	LOX	FN1	SPARC	FLT1	COL5A1	TIMP3	MALAT1	COL3A1
5	GNG11	HNRNPA1	PTMA	PRAC1	TFPI2	TKT	PLAT	IFITM2	TBX3	NPM1
6	KRT19	HMGA1	ACAT2	MT2A	FDPS	ANXA2	FST	CAV1	SLC3A2	RTN4
7	CXCL14	HOPX	IGFBP3	LUM	H19	APCDD1	GBP2	MFAP4	COL14A1	BAMBI
8	INHBA	PRSS35	CCDC80	SERPINE2	FSTL1	SULF1	NPY	NPNT	SPARC	COL4A1
9	MYLK	PTX3	THBS1	ACTA2	CSRP2	TFPI2	TCEAL4	CTHRC1	IGFBP5	IGFBP3
10	CENPF	HMGB2	TOP2A	CKS2	UBE2C	CCNB1	UBE2S	MKI67	ASPM	TPX2
11	HIST1H4C	HIST1H1B	HIST1H1D	DEK	CLSPN	HIST1H1A	ATAD2	H2AFZ	HMGB2	NASP
12	ANKRD1	CCDC146	MEST	OGFRL1	TAGLN	TMEM88	UCP2	OLR1	PRSS35	PDPN
13	CXCL14	H19	COL1A2	DLK1	COL3A1	COL6A1	IGFBP3	APCDD1	BAMBI	COL1A1
14	NPPB	MYL7	EDN1	ANKRD1	TAGLN	CCN2	NPY	FHL1	CCDC80	HHIP
4,8	SPARC	SERPINE2	NEAT1	INHBA	CCDC80	COL4A1	MALAT1	FSTL1	COL11A1	FN1
4,8,14	SERPINE2	INHBA	CCDC80	FSTL1	SPARC	COL4A1	NEAT1	CCN2	SULF1	PRSS35
0,6	KRT19	MT2A	SERPINE1	KRT18	KRT8	PHLDA2	MT1E	ACAT2	HMGA1	CAV1
0,6,12	KRT18	KRT8	KRT19	MT2A	ANKRD1	PHLDA2	MT1E	ACAT2	SERPINE1	GLRX
1,2	S100A6	FTL	BAMBI	CTHRC1	MDK	SELENOM	CRABP2	OST4	SNHG29	RPS10
7,13	CXCL14	H19	IGFBP3	HOPX	APCDD1	DLK1	BAMBI	LUM	MFAP4	COL3A1
1,2,7,13	CXCL14	PCP4	HOPX	FTL	IGFBP3	SERF2	OST4	HSPB6	TIMP1	S100A13
1,2,7,9,13	CXCL14	IGFBP3	BAMBI	CTHRC1	FTL	APCDD1	MDK	LUM	H19	HOPX

### Characteristics of subpopulations

#### Mitotic populations

Evaluation of the expression patterns of cell cycle markers demonstrated that Clusters 3, 10, and 11 were mitotic cell populations (Fig. 2c and Supplementary Table 1). Cluster 10 consisted exclusively of cells in the G2/M phase (99.9%) and had the highest expression of *MKI67* (Supplementary Fig. 4)). Cluster 3 consisted of 80.1% cells in the G2/M phase and 7.8% cells in the S phase, while Cluster 11 consisted of 37.9% G2/M and 62% S phase cells. Cluster 6 had a relatively higher percentage of G2/M and S phase cells (7.6% and 8.7%). The other clusters were mainly composed of G1 phase cells (>92%). Gene ontology (GO) analysis also revealed that Clusters 3, 10, and 11 had characteristics of proliferating cells, such as DNA synthesis and nuclear division (Fig. 3c and Supplementary Data 4).

#### Heart-type group

The clustering results showed that Clusters 4, 8, and 14 were characteristic of CFB (these three clusters accounted for 39.3% of CFB, 12.3% of DFB, and 6.3% of HFB). Therefore, we categorized these clusters as a heart-type group. Analysis of differentially expressed genes (DEGs) showed that *SERPINE2*, *INHBA*, *CCDC80*, *FSTL1*, *and SPARC* were highly expressed in this group (Table 1). Cluster 4 was characterized by high expressions of *COL1A2*, *NEAT1*, *LOX*, *FN1*, and *SPARC* (Table 1 and Supplementary Data 3). Cluster 8 was by those of *INHBA*, *RPSS35*, *CCDC80, SERPINE2*, and *FSTL1*. Cluster 14 was by those of *NPPB*, *MYL7*, *EDN1*, *ANKRD1*, and *TAGLN*. Furthermore, Cluster 14 showed high RGS5 expression, as described above (Supplementary Fig. 4). Trajectory analysis revealed that Cluster 14 was a distinct cell lineage from Clusters 4 and 8 (Fig. 2d, e), suggesting that Cluster 14 is an *RGS5*^+^ subpopulation distinct from Clusters 4 and 8. GO analysis showed that Clusters 4, 8, and 14 were associated with ECM and cytoskeletal organization. In addition, Cluster 4 was associated with lipid and alcohol metabolism (Fig. 3c and Supplementary Data 4).

#### Skin-type group

The clustering results showed that Clusters 0, 6, and 12 were characteristic of DFB (these three clusters accounted for 32.5% of DFB, 22.4% of CFB, and 14.3% of HFB). We categorized these clusters as a skin-type group. DEG analysis revealed that *KRT18*, *KRT8*, *KRT19*, *MT2A*, and *ANKRRD1* were highly expressed in this group (Table 1). Cluster 0 was characterized by high expressions of *KRT8*, *SERPINE1*, *KRT18*, *MT2A*, and *MT1E*. Cluster 6 was by those of *KRT19*, *HMGA1*, *ACAT2*, *MT2A*, and *FDPS*. Cluster 12 was by those of *ANKRD1*, *CCDC146*, *MEST*, *OGFRL1*, and *TAGLN*. GO analysis showed that Clusters 0 and 6 were associated with lipid and alcohol metabolism (Fig. 3c and Supplementary Data 4). Cluster 6 was with nucleotide synthesis, suggesting that this cluster was in the pre-mitotic phase. Cluster 12 was associated with ECM and cytoskeletal organization.

#### Liver-type group and Cluster 5

The clustering results showed that Clusters 1, 2, and 7 were characteristic of HFB (these three clusters accounted for 33.6% of HFB, 18.1% of DFB, and 15.7% of CFB). Cluster 9 was the fourth most cell-rich cluster of HFB (10.0%). Cluster 13, although with fewer cells, was characteristic of HFB (HFB: 6.4%, DFB: 3.5%, CFB: 2.5%). These five clusters accounted for half of the HFB, and we categorized them as a liver-type group.

The DEG analysis showed that *CXCL14*, *IGFBP3*, *BAMBI*, *CTHRC1*, and *FTL* were highly expressed in this group (Table 1). Cluster 1 was characterized by high expressions of *CDKN1A*, *S100A6*, *FTL*, *OST4*, and *MYL9*. Cluster 2 was by those of *S100A6*, *TFPI2*, *LUM*, *MDK*, and *PCOLCE*. Cluster 7 was by those of *CXCL14*, *HOPX*, *IGFBP3*, *LUM*, and *H19*. Cluster 9 was by those of *MYLK*, *PTX3*, *THBS1*, *ACTA2*, and *CSRP2*. Cluster 13 was by those of *CXCL14*, *H19*, *COL1A2*, *DLK1*, and *COL3A1*. When the top 10 DEGs were compared among these five clusters, Clusters 7 and 13 shared five genes (*CXL14*, *IGFBP3*, *H19*, *APCDD1*, *and BAMBI*), and Clusters 1 and 2 did three genes (*S100A6*, *FTL*, *and BAMBI*) (Table 1), indicating that there were three subgroups; Clusters 1 and 2, 7 and 13, and 9. GO analysis showed that Clusters 1 and 7 were functionally different from the other three clusters (Fig. 3c and Supplementary Data 4). Clusters 9 and 13 were associated with ECM and cytoskeleton organization, and Cluster 2 was with nucleotide synthesis.

Cluster 5 contained a similar ratio of CFB, DFB, and HFB and could not be categorized into any group. It was characterized by high expressions of *GNG11*, *HNRNPA1*, *PTMA*, *PRAC1*, and *TFPI2*. This cluster contained a slightly higher percentage of proliferating cells (4.8% S-phase and 3.2% G2/M phase), and the results of trajectory analysis suggested that this cluster was between the mitotic group and Cluster 7 (Fig. 2d, e).

### Regulators of subpopulations

To predict the upstream factors that define individual organ-specific fibroblast models, we analyzed the transcription factor (TF) network using the SCENIC program and identified 248 differentially regulated regulons (Supplementary Fig. 6 and Supplementary Data 5). As shown in a heatmap of the Regulon Specific Score (RSS), each organ-specific fibroblast model had specific TFs under regulation^21^ (Supplementary Fig. 6a). TFs such as PRRX2, HOXB13, MSX2, HOXD10, and HOXA13 for CFB, ZFP64, MEIS1, IRF9, BATF3, and RELB for DFB, and IRX2, HOXB5, POLR3G, KLF9, and KLF2 for HFB were high-scoring regulators (Supplementary Fig. 6c, d). When the top 50 factors (top 20%) were compared, 12 factors were common between DFB and CFB, and one factor was common between DFB and HFB (Supplementary Fig. 6e). In contrast, no factors were common between CFB and HFB. The main regulatory factors for each model consist of different repertoires of factors.

**Fig. 4:**
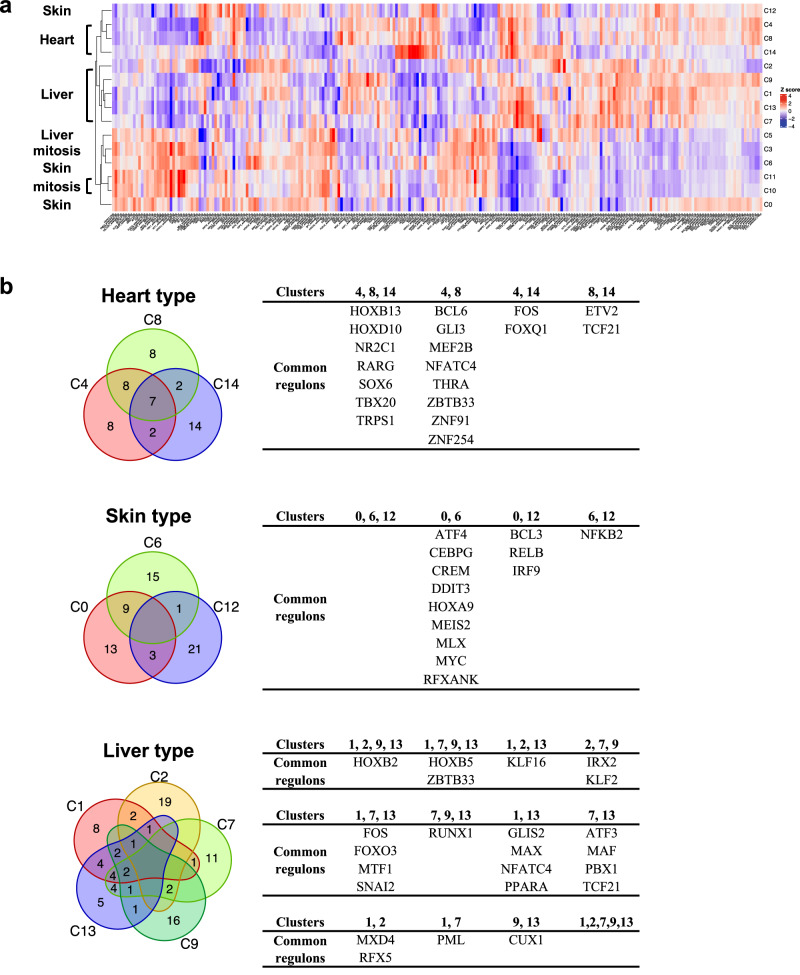
Transcription factor activities in subpopulations. **a** Heatmap showing normalized regulon specific score (RSS) within each cluster. The complete data are presented in Supplementary Data 6. **b** Venn diagrams showing the top 25 (top 10%) regulons in the heart-, skin-, and liver-type populations.

Next, a TF network analysis was performed on the clusters (Supplementary Fig. 7 and Supplementary Data 6). In hierarchical clustering, three major classes were identified: Clusters 4, 8, 12, and 14; Clusters 1, 2, 7, 9, and 13; Clusters 0, 3, 5, 6, 10, and 11 (Fig. 4a). The first class was the heart-type group (Clusters 4, 8, and 14), except for skin-type Cluster 12. The second class consisted exclusively of the liver-type group. The third class was a mixture of the skin-type (Clusters 0 and 6) and mitotic groups.

Among the top 10% of regulons (25/248 factors), seven common TFs, such as HOXB13, SOX6, RARG, HOXD10, TBX20, TRPS1, and NR2C1, were detected in the heart-type group (Fig. 4b). Eight other factors were common to Clusters 4 and 8, suggesting that these two clusters represented a similar subpopulation. The skin-type group had no common factors with any of the top 10% of regulons. Clusters 0 and 6 had nine factors in common, including DDIT3, MYC, and CEBPG, while Clusters 0 and 12 or Clusters 6 and 12 had three or one factor in common, respectively. This suggests that Clusters 0 and 6 are similar. The liver-type group had no common factors. Clusters 1, 2, 9, and 13 had one common factor, HOXB2. Clusters 1, 7, 9, and 13 had two factors, ZBTB33 and HOXB5. Clusters 1 and 13 had 14/25 factors in common, and Clusters 7 and 13 had 11/25 factors in common, but Clusters 1 and 7 had 7/25 factors in common. Cluster 13 may be independently differentiated from Clusters 1 and 7.

### Regulators for myofibroblastic phenotype

Myofibroblasts express high levels of α-smooth muscle actin (ACTA2). High *ACTA2* populations (ACTA2^+^) were identified as Clusters 8 and 14 in the heart-type group, Cluster 12 in the skin-type group, and Cluster 9 in the liver-type group (Fig. 3b). All of these clusters exhibited biological functions such as ECM and cytoskeleton organization by GO analysis (Fig. 3c), suggesting that ACTA2^+^ cells were myofibroblastic-like. Interestingly, Cluster 9 was rarely observed in DFB, whereas Cluster 12 was rarely in HFB (Supplementary Table 1).

**Fig. 5:**
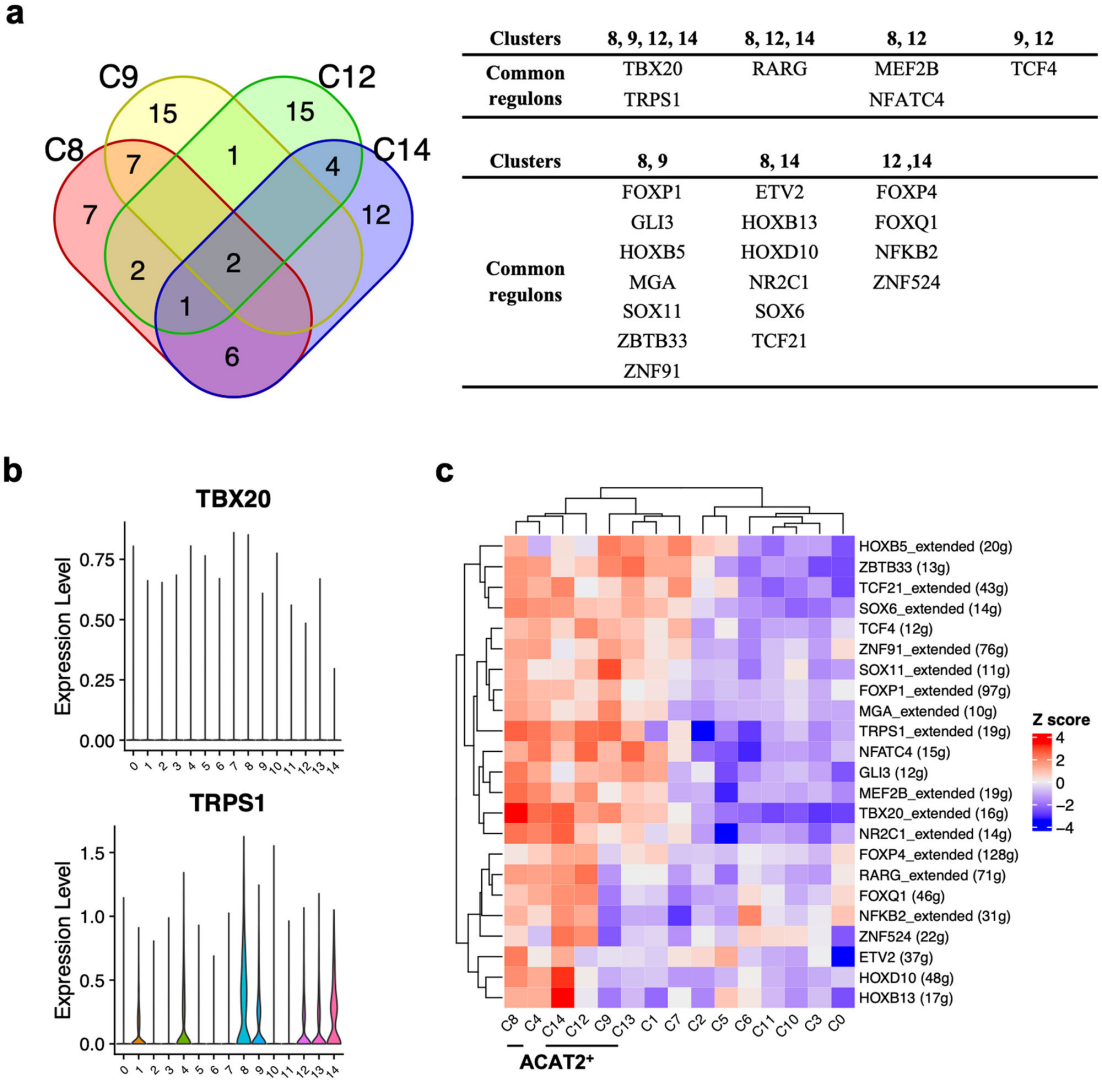
Transcription factor activities in ACTA2^+^ subpopulations. **a** Venn diagrams showing the top 25 (top 10%) regulons in the ACTA2^+^ population. **b** Violin plots indicate the expression levels of *TBX20* and *TRPS1* in the subpopulations. **c** Heatmap showing normalized regulon specific score (RSS) within each cluster.

A survey of ACTA2^+^ regulators indicated that TBX20 and TRPS1 were common regulatory factors in the top 10% of regulators (Fig. 5a). These two factors were also found in the top 10% regulators of Cluster 4, whose *ACTA2* expression was substantially higher but lower than Clusters 8 and 14 in the heart-type group (Fig. 3b). These two factors, therefore, must be associated with the myofibroblast-like properties. The expression level of *TRPS1* was observed in the ACTA2^+^ group, whereas *TBX20* was not (Fig. 5b). Factors common to any of the ACTA2^+^ were ranked low in the ACTA2^−^ group, except for Cluster 4 (Fig. 5c). HOXB5, ZBTB33, TCF21, and SOX6 were found as factors common to both ACTA2^+^ and ACTA2^−^ in liver-type and heart-type groups. Conversely, FOXP4, RARG, FOXQ1, and NFKB2 were as common in heart- and skin-type groups (Fig. 5c). These results suggest that skin-type Cluster 12 and liver-type Cluster 9 were of a distinct ACTA2^+^ subtype.

## Discussion

Understanding the functional differences in fibroblast subpopulations is important for developing therapeutic and preventive methods for diseases associated with fibroblast functions. Despite the research progress, we have not understood the basis of fibroblast heterogeneity among organs. To reveal organ differences in human fibroblasts, we generated three organ-type fibroblasts from iPSC and performed a comparative analysis of their properties. We have found that (1) iPSC-derived fibroblasts using different methods have characteristic organ-type subpopulations that vary in their ratios; (2) These organ-type subpopulations consist of myofibroblast-like ACTA2^+^ and resting-type fibroblasts; (3) ACTA2^+^ subpopulations have distinct characteristics; (4) TBX20 and TRPS1 are common factors in the ACTA2^+^ subpopulation. Our study provides the basis for ontogeny-based processes of fibroblast heterogeneity formation.

Our scRNA-seq analysis revealed 15 clusters from three different organ-specific fibroblast models (Supplementary Fig. 8). These clusters can be categorized into five groups; mitotic-type (Clusters 3, 10, and 11), heart-type (Clusters 4, 8, and 14), skin-type (Clusters 0, 6, and 12), liver-type (Clusters 1, 2, 7, 9, 13), and undetermined (Cluster 5). CFB is composed of 9.4% mitotic-type, 39.3% heart-type, 22.4% skin-type, and 24.2% liver-type. DFB is composed of 24.0% mitotic-type, 12.2% heart-type, 32.5% skin-type, and 23.5% liver-type. HFB is composed of 19.9% mitotic-type, 6.3% heart-type, 14.3% skin-type, and 50.0% liver-type. The percentage of the mitotic-type is likely depending on the experimental conditions. We observed more clusters comprising the liver-type than the other types. This is probably the reason for the high ratio of liver-type in three organ-specific fibroblast models.

The heart-type group contains two subgroups. One is ACTA2^+^ Clusters 8 and 14, and the other is Cluster 4. This cluster appears in transition to Cluster 8, as it exhibits *ACTA2* and the ECM organizing functionalities. The expression patterns of fibroblast marker genes and DEGs are similar between Clusters 4 and 8. Cluster 14 is different from these two clusters and is defined by high expression of *RGS5*, known as a general pericyte marker gene. Although the origin and function of RGS5-expressing fibroblasts have not been determined, overexpression of RGS5 suppresses cardiac hypertrophy and fibrosis in mice and inhibits collagen synthesis in cardiac fibroblasts^22^. Therefore, Cluster 14 is unlikely to participate in cardiac fibrosis. Recent studies have shown that RGS5 is expressed in skin wound fibroblasts and cancer-associated fibroblast (CAF) subpopulations^23–25^. Cluster 14 may share their properties.

The skin-type group shows high expressions of *KRT8*, *KRT18*, and *KRT19*, known as epithelial markers. Cluster 6 appears to be a pre-mitotic type, and Cluster 12 is the active ACTA2^+^ type. Trajectory analysis indicates that Cluster 0 is located in between. In the process of wound healing, dermal fibroblasts proliferate and differentiate into myofibroblasts expressing ECM molecules. Cluster 0 is the most abundant cell population and is likely in transition to both proliferative and ECM synthetic states.

The liver-type contains three subgroups: Clusters 1 and 2, Clusters 7 and 13, and ACTA2^+^ Cluster 9. Clusters 1 and 2 show moderately high expression levels of *ACTA2* but lower expression levels of ECM-organizing genes. In contrast, Cluster 7 and 13 show low expression levels of *ACTA2* but slightly higher expression levels of ECM-organizing genes, especially in Cluster 13. The top DEG of Clusters 1 and 2 are *S100A6*, which is upregulated in myofibroblasts but not in resting hepatic stellate cells (HSCs) in mice^26^. S100A6 activates HSCs and promotes liver fibrosis in mice^27^. Overall expression levels of *S100A6* in the liver-type are higher than in the other groups. Those of *S100A6* are quite low in ACTA2^+^ Cluster 8. These observations suggest that Cluster 8 is unlikely to participate in liver fibrosis. Clusters 7 and 13 have the common top DEG, *CXCL14*. CXCL14-expressing fibroblasts are associated with prostate cancer and Crohn’s disease^28,29^.

ACTA2^+^ myofibroblastic cells are found in each organ-type group and are likely to have different characteristics. Heart-type Cluster 14 has the marked expression of *RGS5*, as mentioned above. In contrast, Heart-type Cluster 8 has high expression of *INHBA* (inhibin subunit beta A), which promotes EMT and fibrosis^30,31^. Cluster 8 also has a high expression of *COL11A1*. Recently, subpopulations with high expression of *INHBA* and *COL11A1* have been identified in CAFs and may be associated with poor prognosis in colorectal cancer^32^. Liver-type Cluster 9 has high expression of PTX3 (pentraxin3), which inhibits inflammation and fibrosis^33–35^. Skin-type Cluster 12 has a high expression of ANKRD1, which facilitates wound healing in skin tissue^36^. Since PTX3 and ANK have opposite functions to each other, the two ACTA2^+^ clusters appear to have opposite properties.

TBX20 and TRPS1 are found as common regulators in the ACTA2^+^ group and Cluster 4. TBX20 is identified as the common regulator in the heart-type group, in agreement with the previous report that TBX20 is a regulator specific to the cardiac system^37^. The expression level of *TRPS1* is higher than that of *TBX20* in the ACTA2^+^ group, suggesting that TRPS1 is an important regulator for myofibroblastic characteristics. A recent multi-omics study has identified TRPS1 as a regulator of regeneration in skin wound healing rather than scar formation^38,39^. This suggests that our identified ACTA2^+^ group is the regeneration-associated fibroblasts rather than myofibroblasts.

The major role of fibroblasts is to form and maintain the ECM. While type I collagen is the major component of collagens, type III collagen is abundant in blood vessels, and type V collagen is often observed in the reticular fibers^40^. The present analysis has found no significant differences in gene expression ratios among the three groups. Fibrillins, components of elastic fibers, have a direct impact on the synthesis of elastic fibers. *FBN1* expression was increased in the CFB, supporting the fact that the lesions of Marfan syndrome caused by *FBN1* are the heart valves and aorta^41,42^. When the ECM is degraded and remodeled, transiently expressed *VCAN* promotes fibroblast proliferation and differentiation into myofibroblasts by enhancing TGFβ-signaling^43^. *VCAN* expression was the highest in the CFB, followed by the DFB and HFB. Our result suggests that the CFB contains more fibrosynthetic cells.

Accumulating studies have illustrated phenotypic diversity within an organ and a tissue, including lung^44^, heart^45,46^, skin^47^, and periodontal tissue^48^. Our results, demonstrating the differences in the expression of ECM molecules, various signaling molecules, and regulon patterns, may provide the basis for organ-specific characteristics in fibrosis, tumor invasion, and other ECM-related pathological conditions.

## Materials and methods

### Cells and culture conditions

The feeder-free human iPSC line, 1383D2, was provided by the RIKEN BRC through the National BioResource Project of the MEXT, Japan^49^. The cells were maintained on laminin-511 (iMatrix511 silk; Matrixome, Suita, Osaka, Japan) coated dish with StemFit AK02N (Ajinomoto, Tokyo, Japan) in a humidified atmosphere of 5% CO_2_ at 37 °C. For subculture, the cells were lifted by Accutase (Innovative Cell Technologies Inc, San Diego, CA) and seeded in the fresh AK02N with 10 µM Y-27632 (Selleck Biotech, Tokyo, Japan). On the next day, the medium was replaced with AK02N without Y-27632.

### Differentiation of human iPSC-derived dermal fibroblasts

Human skin-type fibroblasts were differentiated by a protocol established by Hewitt et al. ^50^ and Kim et al. ^51^. The hiPSC were seeded onto iMatrix511-coated plate in AK02N medium with 10 µM Y-27632. Next days, the cells were cultured in fibroblast differentiation medium 1 (FDM1; 3:1 Dulbecco’s modified eagle’s medium (DMEM; FujiFilm Wako Pure Chemical Co., Osaka, Japan): F12 (FujiFilm Wako Pure Chemical Co.), 5% fetal bovine serum (FBS; Merck KGaA, Darmstadt, Germany), 0.18 mM adenine (FujiFilm Wako), 10 ng/ml human epidermal growth factor (EGF; FujiFilm Wako Pure Chemical Co.), and 5 µg/ml human insulin (FujiFilm Wako Pure Chemical Co.) for 4 d. Then the cells were cultured in FDM1 containing 0.5 nM human bone morphogenetic protein 4 (BMP4; Reprocell Japan, Yokohama, Japan) for 3 d. At day 7, the cells were passed onto Geltrex-coated plate (ThermoFisher Scientific K.K., Tokyo, Japan) and cultured in FDM2 (1:1 DMEM: F12, 5% FBS and 1% non-essential amino acids (ThermoFisher Scientific K.K.)) for 7 d. At day 14, the cells were passaged onto a non-coated culture plate in EDM1 for 7 d. At day 21, the cells were passaged onto type-I collagen (Col-I; Nitta gelatin Inc., Osaka, Japan) coated dishes in EDM1 for a few days. The differentiated DFB were cultured in FibroGRO medium (Merck KGaA) on Col-I-coated dish.

### Differentiation of human iPSC-derived cardiac fibroblasts

Epicardiac fibroblasts as the CFB were differentiated by a protocol established by Bao et al.^52^. The hiPSC were seeded onto an iMatrix511-coated plate in AK02 medium with 10 µM Y-27632. The next day, the cells were treated with 6 µM CHIR99021 (Selleck Biotech) in RPMI/B27 (RPMI (FujiFilmWako Pure Chemical Co.) containing with B27 supplement minus insulin (ThermoFisher Scientific K.K.)) for 24 h and then treated with RPMI/B27 for 2 d. At day 3, the cells were treated with 5 µM IWP-2 (Nacalai Tesque Inc., Kyoto Japan) in 1:1 conditioned media to fresh RPMI/B27. After 2 d (day 5), the cells were treated with RPMI/B27. On day 6, cardiac progenitors were detached by Accutase and seeded onto a Geltrex-coated plate in LaSR medium (Advanced DMEM/F12 (ThermoFisher Scientific K.K.) containing 1.3% GlutaMax (ThermoFisher Scientific K.K.) and 0.1 mg/ml ascorbic acid). Then, the cells were treated with 3 µM CHIR99021 in LaSR medium for 2 d and were cultured in LaSR for 3 d. At day 12, the cells (epicardial cells) were detached by Accutase and seeded onto a Geltrex-coated plate in LaSR medium with 0.5 µM A83-01. To differentiate epicardiac fibroblasts, the cells were cultured in LaSR with 5 ng/ml human basic fibroblast growth factor (bFGF; Reprocell Japan)) and 0.5 µM A83-01 (Merck KGaA) for 10 d. The differentiated CFB were cultured in FibroGRO medium on a Col-I-coated dish.

### Differentiation of human iPSC-derived hepatic fibroblasts

Hepatic stellate cells as the HFB were differentiated by a protocol established by Miyoshi et al. ^53^. The hiPSC were seeded onto an iMatrix511-coated plate in AK02 medium with 10 µM Y-27632. The next day, the cells were incubated with Advanced-DMEM/F12 containing 2% B27 supplement, 1% N2 supplement (FujiFilm Wako Pure Chemical Co.), 10 µM CHIR99021, 10 ng/ml bFGF, 30 ng/ml BMP-4, and 10 ng/ml human activin A (API Co., Ltd, Gifu, Japan) for 3 d. Next, the cells were incubated with Advanced-DMEM/F12 containing 2% B27 supplement, 1% N2 supplement, 100 ng/ml bFGF, and 50 ng/ml BMP-4 for 3 d. The differentiated HSCs were cultured in FibroGRO medium on a Col-I-coated dish.

### Quantitative real-time PCR

Total RNAs were extracted by RNeasy plus mini kit (QIAGEN K.K, Tokyo Japan) and cDNAs were synthesized with ReverTraAce qPCR RT master mix with gDNA Remover (Toyobo Co. Ltd., Osaka, Japan). Quantitative real-time PCR (qRT-PCR) was performed on a QuantStudio 3 Real-time PCR system (ThermoFisher Scientific K.K.) using iTaq Universal SYBR Green Supermix (Bio-Rad Laboratories Inc., Hercules, CA). The PCR primer sets are represented in Supplementary Table 2. The reaction was performed for 40 cycles of 95 °C for 15 s and 60 °C for 60 s followed by 95 °C for 10 min. The expression level was analyzed by the comparative Ct method and normalized to that of glyceraldehyde-3-phosphate dehydrogenase (GAPDH).

### Analysis of Single cell RNA sequencing data

Rhelixa, Inc. (Tokyo, Japan) performed the single cell RNA library preparation by 10× chromium, sequencing by Illumina NovaSeq 6000, and data analysis by Cell Ranger v6.1.2. Further analysis of the raw data was performed with Seurat package v4.4.0^54^. For quality control, we first filtered the cells with a threshold of over 2500 “nFeature_RNA”, 5000 to 200,000 “nCount_RNA”, and less than 10 “percent.mt”. The data of total 5515 cells of DFB, 4490 cells of CFB, and 5799 cells of HFB were normalized using the Log Normalization method and integrated with 2000 variable integration features. After merging the Seurat Objects, the data was scaled with the *ScaleData* function and identified the principal component analysis (PCA, npcs = 30). A clustering and dimensionality reduction of UMAP was performed using the *FindNeighbours*, *FindClusters*, and *RunUMAP* functions, respectively (dims = 1:15 and resolution = 1.2). Cell cycle scoring was performed with the *CellCycleScoring* function. The differentially expressed genes (DEGs) of each cluster were identified using the *FindAllMarkers* function (min.pct = 0.25 and logfc.threshold = 0.25). The trajectory analysis was performed using Monocle 3 package 1.3.1^55^. Gene ontology analysis was performed using clusterProfiler package 4.6.2^56^. Single cell regulatory network inference and clustering (SCENIC) package 1.3.1 were used to infer gene regulatory networks and the key transcription factors of subpopulations^57^. Venn diagrams were drawn by using Venn package 1.11. Heatmaps were created with ComplexHeatmap package 2.14.0.

### Statistics and reproducibility

Statistical analyses were performed using IBM SPSS statistics 28 (IBM Corp, Armonk, NY). One-way ANOVA followed by Dunnett’s or Tukey’s multiple comparison test was used, and *p*-values < 0.05 were considered significant.

### Supplementary information

Supplemental information

Description of Additional Supplementary Files

Supplemental Data 1

Supplemental Data 2

Supplemental Data 3

Supplemental Data 4

Supplemental Data 5

Supplemental Data 6

Supplemental Data 7

## Data Availability

Single cell RNA-seq data in this study are available at Gene Expression Omnibus (GEO) with accession number GSE244766. Primary fibroblast datasets were available at GEO with accession numbers GSM5104820 (PCS-201-012)^17^, GSM6894025 (BJ)^18^, and GSM6894025 (CCD-18Co)^19^. Human 15 organs datasets were available at GEO with accession number GSE159929 or at the Human Cell Atlas (https://explore.data.humancellatlas.org/projects/376a7f55-b876-4f60-9cf3-ed7bc83d5415)^20^. Source data are provided in Supplementary Data 7.
