# Supplementary material for: Subpopulations of fibroblasts derived from human iPS cells
Source: Commun Biol. 2024 Jun 18;7:736. doi: 10.1038/s42003-024-06419-8 (PMC11189496; doi:10.1038/s42003-024-06419-8)
Supplement: Supplementary file 1 — Supplemental information [file 42003_2024_6419_MOESM1_ESM.pdf]

Supplementary Table 1. Proportions of cells in the clusters.

		Ratio and counts in sample*				Cell cycle score$		
Cluster	Type	CFB	DFB	HFB	Total	G1	S	G2M
0	Skin	9.80%	14.90%	8.30%	11.00%	95.93%	3.04%	1.03%
		440	823	480	1743	1672	53	18
1	Liver	8.50%	4.80%	10.90%	8.10%	95.16%	1.17%	3.67%
		382	266	632	1280	1218	15	47
2	Liver	5.10%	7.10%	11.30%	8.10%	97.02%	2.19%	0.78%
		227	392	657	1276	1238	28	10
3	Mitosis	4.10%	9.40%	9.60%	8.00%	11.56%	7.76%	80.68%
		186	518	559	1263	146	98	1019
4	Heart	11.60%	8.50%	4.10%	7.80%	97.16%	1.62%	1.22%
		523	470	240	1233	1198	20	15
5	ND	4.60%	7.80%	9.40%	7.50%	91.96%	4.82%	3.21%
		208	428	546	1182	1087	57	38
6	Skin	7.50%	8.80%	5.30%	7.20%	83.72%	8.67%	7.61%
		338	486	306	1130	946	98	86
7	Liver	2.20%	6.20%	11.40%	7.00%	97.18%	2.82%	0.00%
		98	341	661	1100	1069	31	0
8	Heart	15.30%	3.40%	1.70%	6.10%	95.05%	2.58%	2.37%
		686	188	96	970	922	25	23
9	Liver	5.90%	1.90%	10.00%	6.00%	96.62%	3.06%	0.32%
		264	103	581	948	916	29	3
10	Mitosis	2.30%	7.60%	5.20%	5.20%	0.00%	0.12%	99.88%
		105	420	302	827	0	1	826
11	Mitosis	3.00%	7.00%	5.10%	5.10%	0.12%	61.99%	37.88%
		136	384	293	813	1	504	308
12	Skin	5.10%	8.80%	0.70%	4.80%	94.86%	2.50%	2.64%
		230	486	43	759	720	19	20
13	Liver	2.50%	3.50%	6.40%	4.30%	98.37%	1.18%	0.44%
		111	191	374	676	665	8	3
14	Heart	12.40%	0.30%	0.50%	3.80%	97.02%	2.48%	0.50%
		556	19	29	604	586	15	3

*Ratio of clusters in the sample (counts). $Ratio of cell cycle phases in clusters. ND: not determined.

Supplementary Table 2. Primers for qRT-PCR.

Target Genes	Forward Primer	Reverse Primer
ACTA2	5'-GCGTGGCTATTCCTTCGTTA-3'	5'-ATGAAGGATGGCTGGAACAG-3'
COL1A1	5'-TCTGCGACAACGGCAAGGTG-3'	5'-GACGCCGGTGGTTTCTTGGT-3'
COL3A1	5'-CCAGGAGCTAACGGTCTCAG-3'	5'-CAGGGTTTCCATCTCTTCCA-3'
DCN	5'-TGCTGTTGACAATGGCTCTC-3'	5'-CCTTTTTGGTGTTGTGTCC-3'
GAPDH	5'-GTCTCCTCTGACTTCAACAGCG-3'	5'-ACCACCCTGTTGCTGTAGCCAA-3'
NANOG	5'-CTCCAACATCCTGAACCTCAGC-3'	5'-CGTCACACCATTGCTATTCTTCG-3'
OCT3/4	5'-CCTGAAGCAGAAGAGGATCACC-3'	5'-AAAGCGGCAGATGGTCGTTTGG-3'
PDGFRA	5'-AACCCTGCTGATGAAAGCAC-3'	5'-TCCTTTCTAGCATGGGGACA-3'
POSTN	5'-GACCGTGTGCTTACACAAATTG-3'	5'-AAGTGACCGTCTCTTCCAAGG-3'
VIM	5'-AGGCAAAGCAGGAGTCCACTGA-3'	5'-ATCTGGCGTTCCAGGGACTCAT-3'

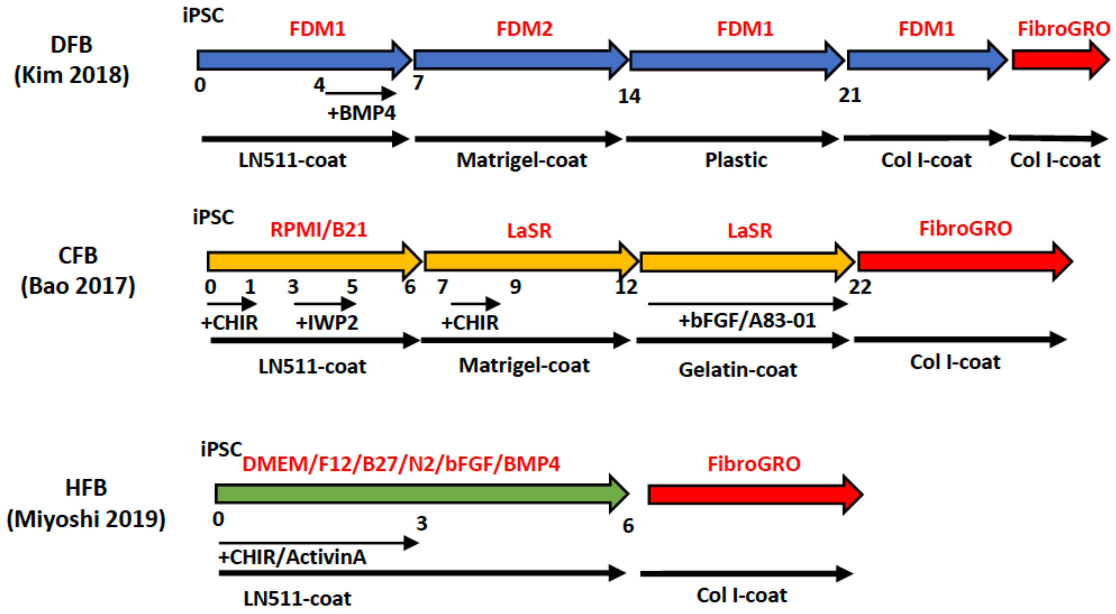

Supplementary Figure 1. Schematic of the fibroblast differentiation process.

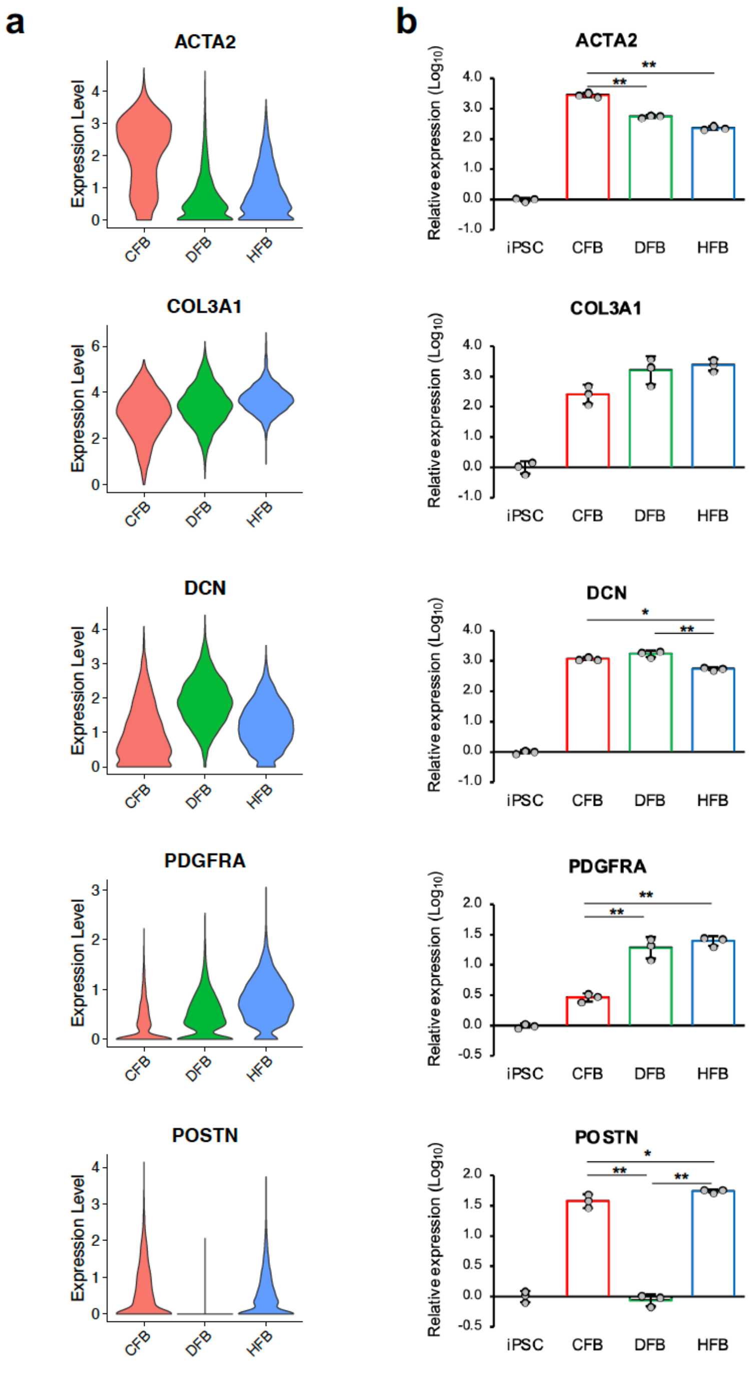

Supplementary Figure 2. Expression levels of fibroblast marker genes in differentiated fibroblasts.

a) Violin plots indicate expression levels of fibroblast marker genes. **b**) Expression levels of fibroblast marker genes were examined using qRT-PCR. Data represent the mean ± SD of three independent experiments. Significance was determined using ordinary one-way analysis of variance (ANOVA) with Tukey's multiple comparison test (* $p < 0.05$, ** $p < 0.01$).

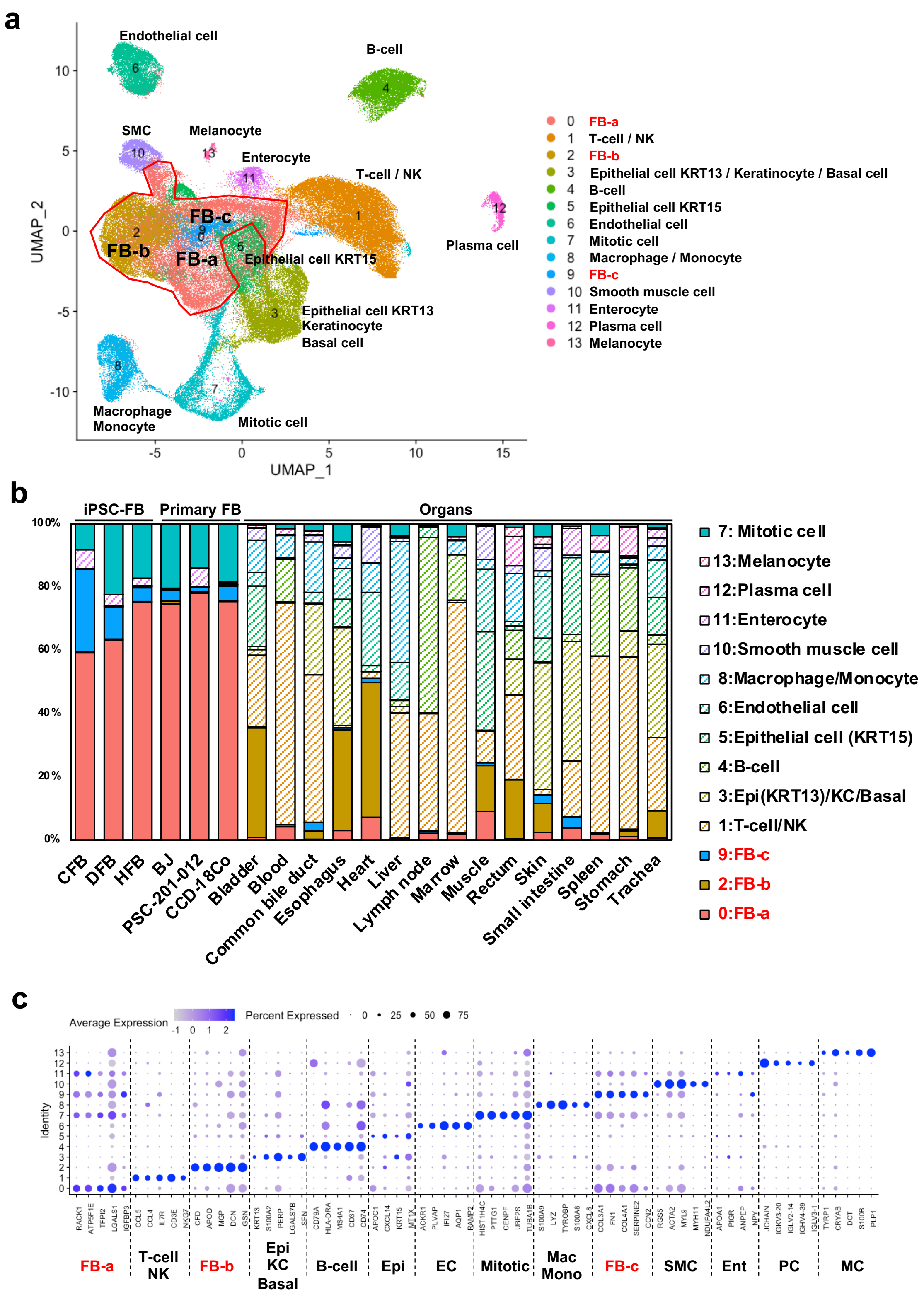

Supplementary Figure 3. Analysis of scRNA-seq data from differentiated fibroblasts and public databases.

scRNA-seq data from three differentiated fibroblasts and data from PCS-201-012 (GSM5104820),

BJ (GSM6894025), CCD-18Co (GSM6894025), and 15 human organs (GSE159929) were integrated and analyzed by Seurat. For quality control, PCS-201-012 and BJ data were filtered with a threshold of >2,500 nFeature_RNA, 5,000–200,000 nCount_RNA, and less than 10 percent.mt. CCD-18Co data were filtered using a threshold of >2,500 nFeature_RNA, 5,000–200,000 nCount_RNA, and less than 15 percent.mt. **a)** UMAP plot analysis (dims = 1:17, resolution = 0.3). **b**) Bar graph showing the proportion of clusters in each sample. The raw data are presented in Supplementary Data 2. **c**) Dot plot showing the expression of the top five DEGs. The complete DEG data are presented in Supplementary Data 1. NK, natural killer cell; Epi, epithelial cell; KC, keratinocyte; Basal, basal cell; EC, endothelial cell; Mitotic, mitotic cell; Mac, macrophage; Mono, monocyte; SMC, smooth muscle cell; Ent, enterocyte; PC, plasma cell; MC, melanocyte.

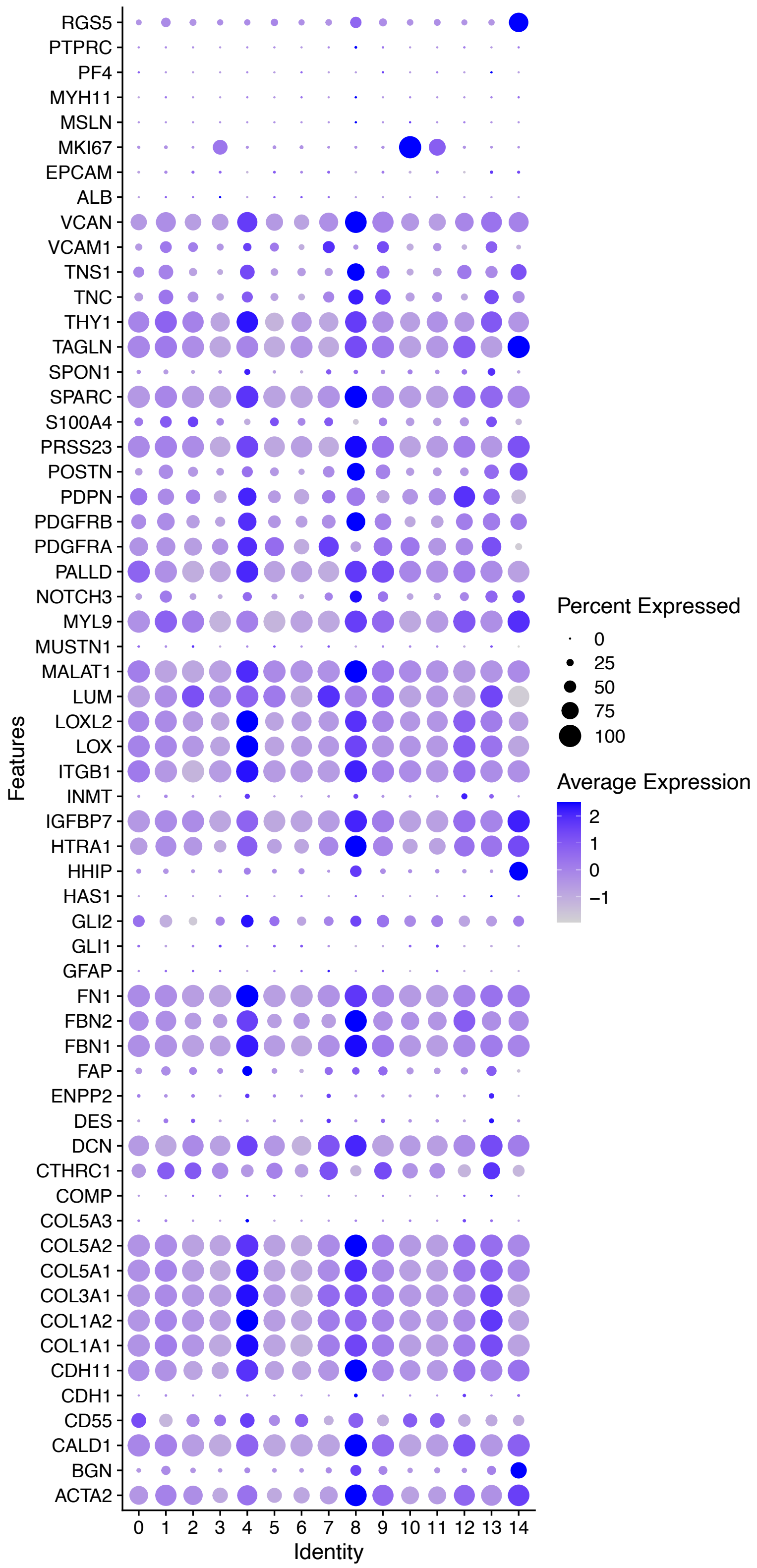

Supplementary Figure 4. Dot plot showing expressions of fibroblast marker genes.

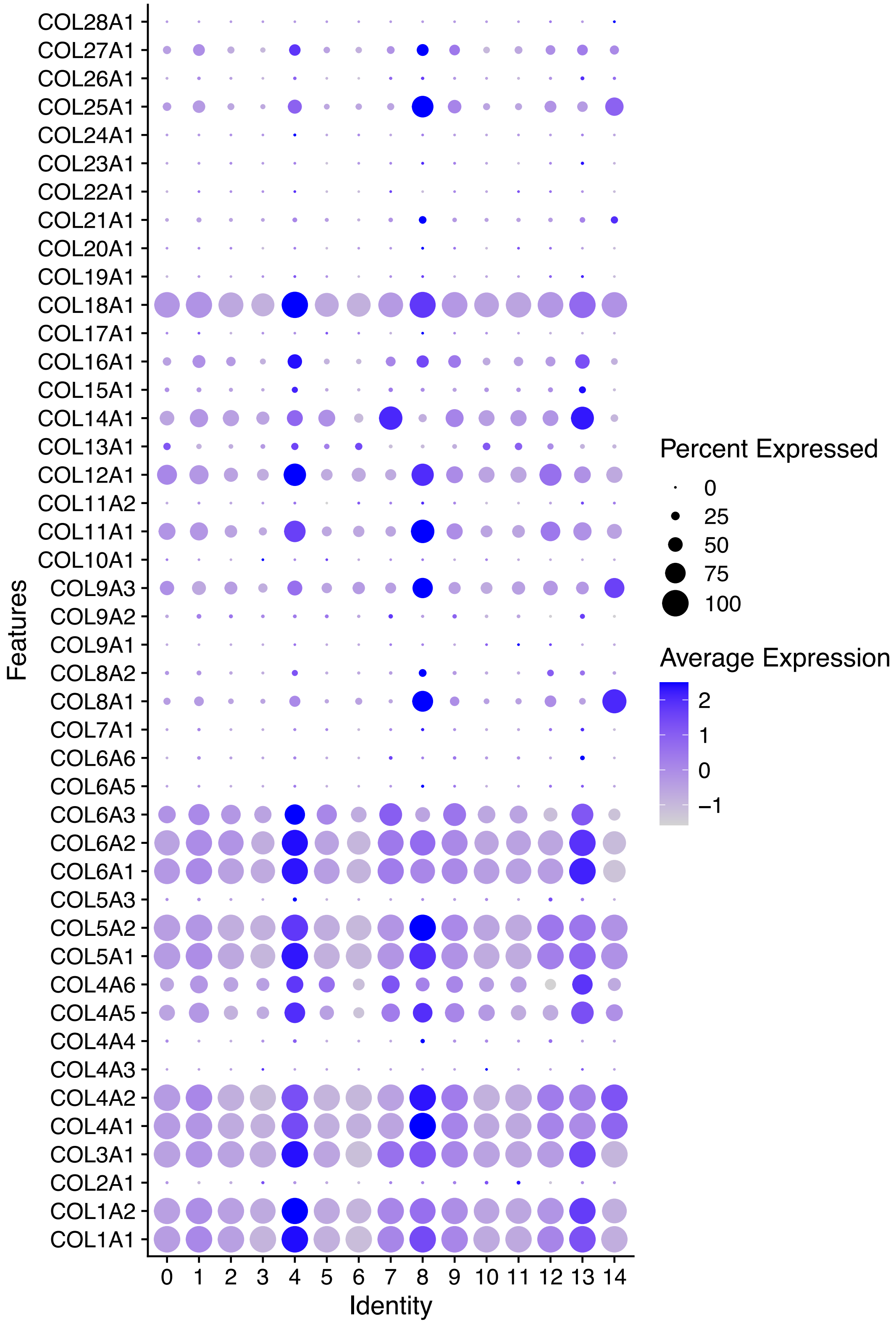

Supplementary Figure 5. Dot plot showing expressions of collagen genes.

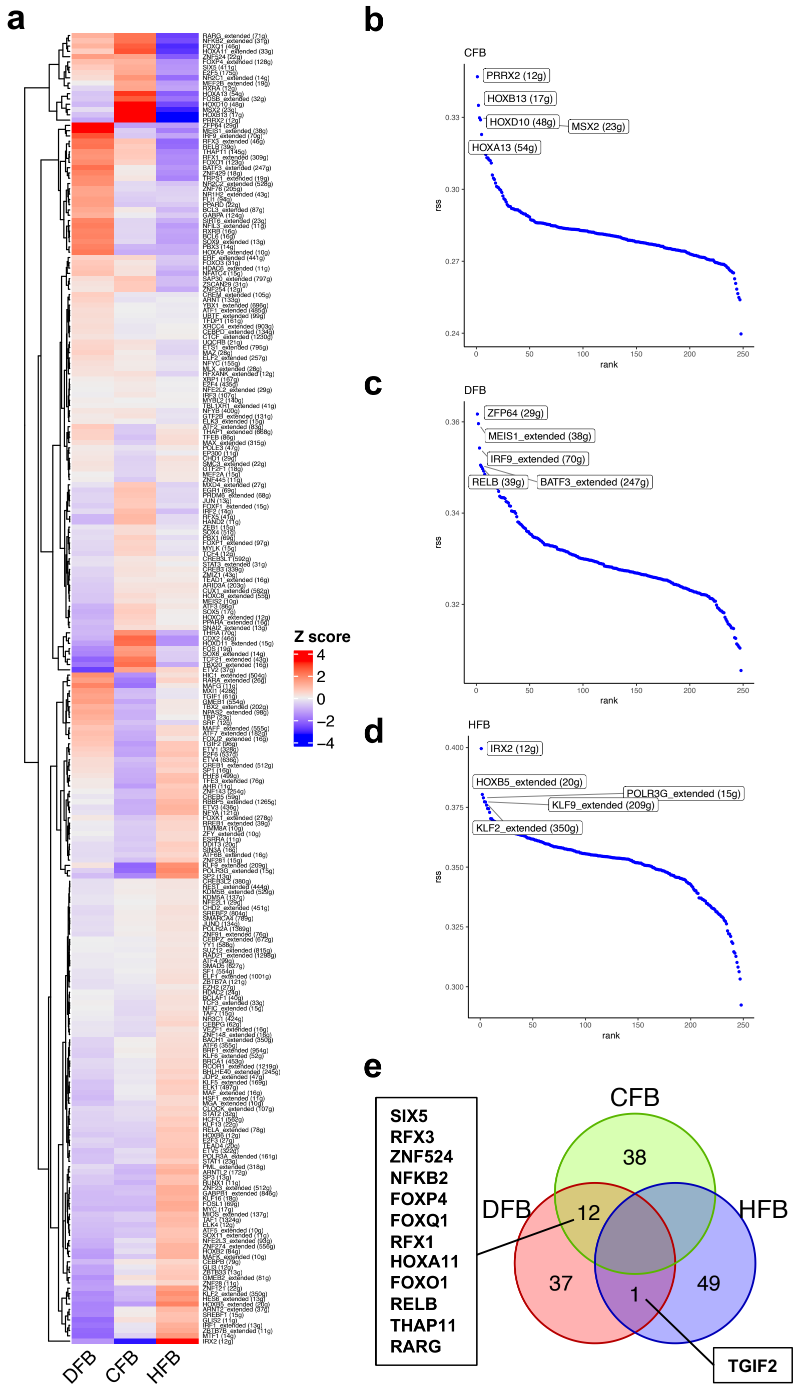

Supplementary Figure 6. Transcription factor activities in the differentiated fibroblasts.
a) Heatmap showing normalized regulon activity in fibroblasts. The complete data are presented in Supplementary Data 5. **b, c, d)** Regulon specificity scores in CFB (**b**), DFB (**c**), and HFB (**d**) with the top five specific TFs labeled. **e**) Venn diagrams showing the top 50 regulons in fibroblasts.

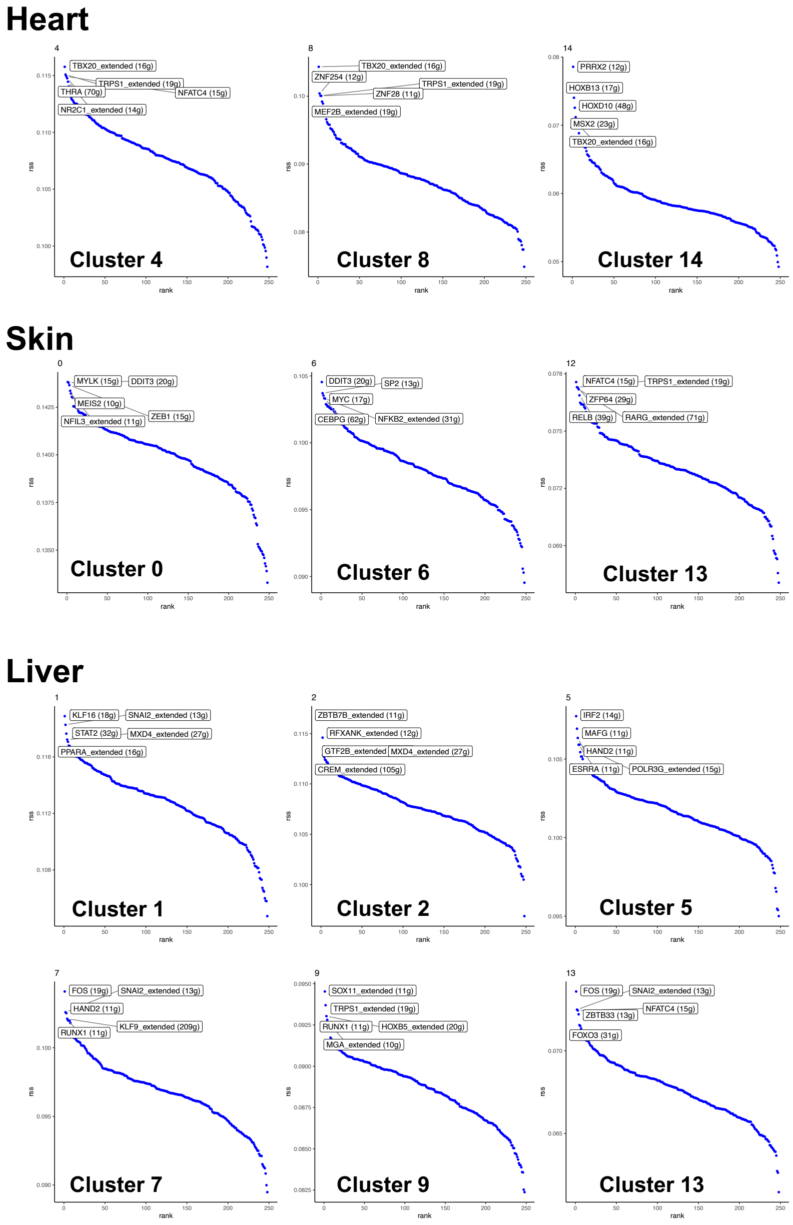

Supplementary Figure 7. Regulon specificity score in subpopulations labeled with the top five specific TFs.

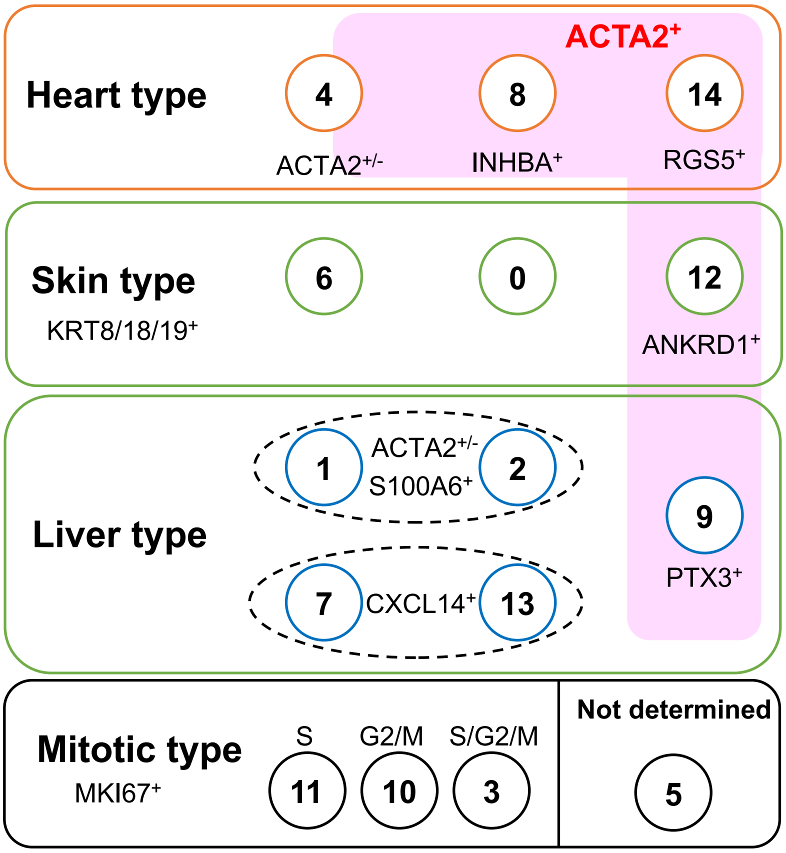

Supplementary Figure 8. Summary of subpopulations.
