# Supplementary material for: Subpopulations of fibroblasts derived from human iPS cells
Source: Commun Biol. 2024 Jun 18;7:736. doi: 10.1038/s42003-024-06419-8 (PMC11189496; doi:10.1038/s42003-024-06419-8)
Supplement: Supplementary file 2 — Description of Additional Supplementary Files [file 42003_2024_6419_MOESM2_ESM.pdf]

Description of Additional Supplementary files

File name: Supplementary Data 1

Description: The results of differentially expressed genes analysis in primary fibroblasts and 15 organs

File name: Supplementary Data 2

Description: Proportions of cells in the clusters in primary fibroblasts and 15 organs

File name: Supplementary Data 3

Description: The results of differentially expressed genes analysis in iPSC-derived fibroblasts

File name: Supplementary Data 4

Description: The results of gene ontology enrichment analysis of biological processes

File name: Supplementary Data 5

Description: The results of regulon analysis and regulon specific score in the fibroblast models.

File name: Supplementary Data 6

Description: The results of regulon analysis and regulon specific score in the clusters

File name: Supplementary Data 7

Description: The source data behind the graphs in Figure 1b and Supplementary Figure 2b.
